# Age-related changes in visuo-proprioceptive processing in perceived body position

**DOI:** 10.1038/s41598-022-12022-w

**Published:** 2022-05-18

**Authors:** Wataru Teramoto

**Affiliations:** Department of Psychology, Graduate School of Humanities and Social Sciences, Kumamoto University, 2-40-1 Kurokami, Kumamoto City, 860-8555 Japan

**Keywords:** Human behaviour, Perception, Cognitive ageing

## Abstract

This study investigated age-related change in visuo-proprioceptive processing in the perceived body position using mirror hand/foot illusions, focusing on its temporal characteristics, its dependency on body parts, and its association with older adults’ fall risk. Either immediately or 15 s after the exposure to the mirror-induced inconsistency of visuo-proprioceptive signals regarding the right hand or foot position, participants performed a reaching task using the unseen, illusion-affected hand or foot. Results showed clear age group differences. Specifically, older adults exhibited larger reaching errors than younger adults in the hand condition, and after the 15 s delay in the foot condition. Further, the reaching errors were constant across time for older adults but decreased after the delay in young adults, regardless of the tested body part. Particularly, older adults’ risk of falling, which was assessed by the timed up-and-go test, was negatively correlated with the reduction of reaching error across time. This suggests that older adults, especially those with a high risk of falling, face difficulties in appropriately processing visual and proprioceptive information for body perception in accordance with their external environment.

## Introduction

Visual and proprioceptive signals play a fundamental role in perceiving current body positions in space and, consequently, affect the control of body movements, as demonstrated by studies using prisms^1^, artificial body parts^2^, mirrors^3^, and video systems, including virtual reality systems^4^. Spatial inconsistencies between these senses can cause the perceived position of the body to be biased in the direction indicated by the visual system. In typical rubber hand experiments, for example, the perceived position of the occluded real hand is shifted toward the seen rubber hand placed nearby. Studies suggest that visual and proprioceptive information is integrated based on the relative reliability of the information in the perception of body positions^5,6^, similar to the other types of multisensory processing such as audiovisual^7^ and visuotactile perception^8^.

Age seemingly affects multisensory processing and the processing of each single sensory modality. Compared to young adults, older adults exhibit a greater influence of multisensory presentation over unisensory presentation, for example, in stimulus detection tasks^9,10^, maybe due to degradation of each sensory modality, slowness of processing speed, attentional deficits, increased levels of internal noise, and so on. Regarding the perceived position of body parts, however, most studies using rubber hand illusions show no or little effect of age^11–15^. One study that shows age-related changes in the perceived position of the real hand—increased drift toward the rubber hand with an increase in age—had participants below 60 years old^14^. Conversely, literature not focusing on rubber hand illusions showed that distorted visual signals strongly biased reaching performance in older adults compared with younger adults, suggesting that older adults rely more on visual than proprioceptive information in perceiving hand position^16–18^. Thus, the rubber hand procedure used in the aforementioned studies is perhaps not sensitive enough in unveiling the age-related changes in the perceived position of the body parts.

**Figure 1:**
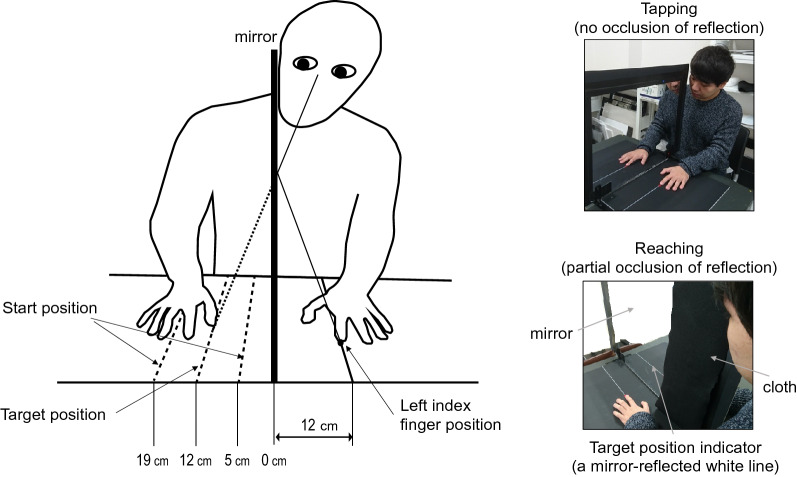
An experimental setup for the mirror hand condition. A mirror was positioned vertically in the middle of a table, with its reflective surface facing the participant’s left. The participant’s left hand was placed at 12 cm left from the mirror where a small red sticker was placed. Participants were asked to synchronously tap both index fingers while viewing the mirror-reflected hand to induce the mirror illusion (upper right panel). The start position of reaching by the unseen, right hand was 19 cm to the right of the mirror for the immediate and delay conditions, and either 5 cm or 19 cm to the right of the mirror for the baseline condition. During reaching, participants were asked to reach the target line (12 cm right from the mirror) indicated in the mirror, which was actually the mirror-reflection of a line drawn 12 cm left from the mirror (bottom right panel). Note that the mirror-reflection of the left hand was occluded by a small cloth during reaching.

Therefore, this study aimed to further investigate the age-related changes in visuo-proprioceptive processing in the perceived body position. Here, the mirror hand illusion procedure was used^3^ instead of the rubber hand procedure to exclude the effects of the appearance of rubber hands. Studies show that differences between the real and rubber hands can reduce the feeling that the rubber hand is ones’ own^19^. This might hinder the age-related changes in the visuo-proprioceptive processing in the perceived body position from being pronounced. In the typical mirror hand illusion procedure, a mirror is positioned vertically in the middle of a table. Participants place their hands on each side of the table sectioned by the mirror (Fig. 1). On the reflective surface side, participants can see one of their hands and its mirror reflection. After several seconds of synchronous tapping of both hands while viewing the mirror-reflected hand, participants feel the mirror-reflected hand as the unseen, opposing hand. In case spatial inconsistency is introduced between the mirror-reflected and unseen hands, reaching with the unseen hand is biased toward the position specified by the mirror-reflected hand^3,20,21^. This study adapted the aforementioned typical procedure. As the mirror-reflected hand is the participants’ own hand, the appearance differences were less than those in the rubber hand experiment. Further, in the typical mirror hand procedure, both illusion induction and responses are made by the participants’ own movements. Previous studies show that the shift of perceived body position was more salient in manual responses than in perceptual responses when illusory perception of the hand was induced by the participants’ own movements, while the relationship was reversed when it was induced by passive movements or visuotactile synchronous stimulation^22–24^. Manual responses include reaching or pointing by the participants’ own hand, while perceptual responses include a ruler technique, where participants give their answers based on the numbers on a ruler placed on the participants’ hidden hand, or cursor navigation, where participants stop a moving cursor when it reaches the perceived body position. Thus, these studies suggest that the consistency in modality used in illusion induction and response can influence the magnitude of the illusion. Hence, this mirror hand procedure may be more susceptible to age effects on the perceived body position than the aforementioned mirror hand procedure.

This study focused on two aspects. First, the effect of temporal lag after visual information was occluded. Bellan et al.^4^ investigated how the contribution of visual and proprioceptive information to the perceived hand position changed over time. They asked participants to observe their own hands through a real-time video system and maintain their hand positions within a specific area which was changed over time. The video image of one hand was independently shifted from its actual position without bringing it to the notice of the participants, so that the discrepancy between the seen and felt hand positions could be yielded. After exposure to this situation, the video image of the hand was hidden. Then, the perceived hand position was measured over time by asking the participants to stop a horizontally-moving visual arrow when it was aligned with the felt hand position. The results showed that the perceived position of the unseen hand was initially displaced to the last seen position but shifted to the physically correct position over time. They suggest that the reliability of remembered visual information degrades over time relative to that of proprioceptive information such that the perceived position of the unseen hand moved to the physical position. This drives me to ask whether such temporal characteristics can be observed in older adults. How visuo-proprioceptive processing in perceived hand position changes over time in older adults has not been investigated. However, previous studies have shown that, unlike younger adults, older adults cannot quickly change multisensory processing in reaching^25^ and postural control^26,27^ depending on changes in the surrounding environment. Thus, it is likely that older adults’ perceived body position changes less over time than younger adults.

Second, this study focused on the differences between body parts, specifically, the perceived body position with respect to the hand and foot. Studies show that the body ownership illusions, similar to the rubber hand illusion, can extend to the foot^28,29^ and other body parts^30,31^. Generally, with age, proprioception in distal body parts is more impaired than that in proximal body parts^32^. Further, proprioceptive information from the lower limb may deteriorate more than that from other body parts because studies have shown a decrease in gait function with increasing age^33^. Recently, Hide et al.^34^ demonstrated that while young and older adults did not differ either in rubber hand or foot illusions at a group level, older adults exhibited a distinctive feature in multisensory processing in body ownership of the foot but not the hand. Specifically, for the foot, older adults with a lower risk of falling, measured by the Timed Up and Go (TUG) test, hardly experienced the ownership illusion, while those with a higher risk of falling experienced it with shorter latency and no weaker than the younger adults. Several studies reported that older adults with higher risk of falling or a history of falling were more susceptible to the sound induced flash illusion^35^ or visuotactile interaction^36^ than those with a lower risk of falling. These studies suggest some association between older adult’s multisensory processing and physical and/or cognitive functions related to fall, although the underlying mechanisms remain unclear. Thus, the visuo-proprioceptive processing in the perception of body position could possibly change depending not only on body parts but also older adults’ risk of fall. This study exploratorily investigated this point.

## Methods

### Experimental design

There were three factors in this study: Age group (younger and older adults), Body part (hand and foot), and Reaching type (immediate, delay, and baseline). Age was a between-participants factor, and others were within-participant factors. Each body part condition was conducted in a different session. The order of the hand and foot sessions was counterbalanced across participants. In Reaching type, the immediate and delay conditions were defined by the temporal lag from the mirror illusion induction (10-times synchronous tapping with both hands or feet). Reaching was soon after the illusion induction in the immediate condition and 15 s later in the delay condition (Table 1). A single illusion induction was followed by one reaching condition (i.e., not by two reaching conditions in sequence). The start and target positions were fixed across these conditions—19 cm and 12 cm right from the mirror, respectively. Four trials were performed for each reaching timing per body part. The reaching conditions order was counterbalanced across participants within each body part condition. Before these, the baseline condition, where no illusion induction procedure was applied, was conducted because reaching itself could differ between young and older adults. The baseline condition had four trials; all the trials were first conducted in each body part session. The baseline had two start positions (5 cm or 19 cm right from the mirror; which were alternately conducted), not one (different from the other reaching condition), to make participants believe that the start position was changeable in the following trials.

**Table 1:** Experimental parameters for each reaching type.

Reaching type	Illusion induction	Delay	Start position
Immediate	Yes	0 s	19 cm
Delay	Yes	15 s	19 cm
Baseline	No	–	5 cm, 19 cm

### Participants

In Bellan et al.^4^, there were 16 (Experiment 1) and 18 (Experiment 2) participants. However, there were no previous data regarding the aging effect. As a reference, the sample size was calculated using G*power^37^ with the medium-level effect (*f* = 0.25). The required total sample size (parameters: α = 0.05, 1 − β = 0.8; repeated measures, within-between interaction) was 14 for each older and young adult group. Accordingly, 17 community-dwelling older adults (mean age: 75.29 ± 5.03 years, minimum = 70, maximum = 85; six men) were recruited from the senior participant database in my laboratory. They self-reported no dementia, depression, stroke, parkinsonism, or orthopedic diseases and were not currently receiving treatment with neuroleptics. All participants scored more than 27 on the Mini-Mental State Examination (mean score: 29.35 ± 0.86)^38^. None reported defects in vision in either eye (e.g., macular degeneration, cataract, or glaucoma). All participants’ visual acuity, equivalent to the reciprocal of the minimum resolvable visual angle, was assessed at viewing distances of 0.4 m and 5 m with both eyes open using Landolt C charts using visual acuity correction by convex or bifocal glasses. The mean visual acuities (± standard deviation) of near and far vision were 0.77 (± 0.19) and 0.95 (± 0.19), respectively. Young adult participants were 26 introductory psychology students at Kumamoto University (mean age: 19.58 ± 1.24 years, minimum = 18, maximum = 22; 7 men). All participants voluntarily enrolled in this study and provided written informed consent for participation and to publish their accompanying images in an online publication before commencement. Although the number of those enrolled was larger than that of older participants, I did not narrow down the list of participants. They had normal or corrected-to-normal visual acuity. Their mean visual acuity of far vision was 1.23 (± 0.40) (near vision was not measured for young adults). This study was conducted according to the principles of the Declaration of Helsinki and was approved by the Ethics Committee of the Graduate School of Social and Cultural Sciences, Kumamoto University.

### Apparatus and materials

**Figure 2:**
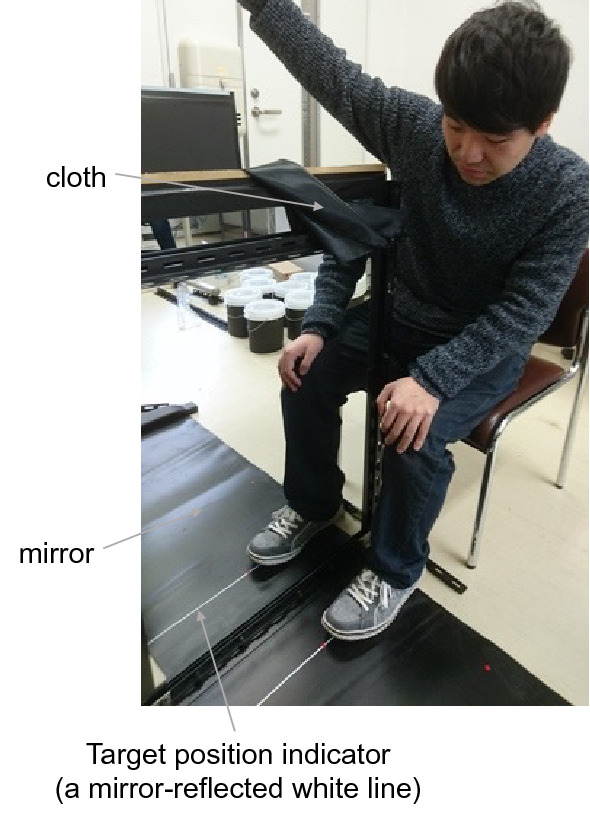
An experimental setup for the mirror foot condition. A mirror was positioned on the floor between the participant’s legs.

For the hand condition, a 60 cm × 45 cm mirror was positioned vertically in the middle of a table, with its reflective surface facing the participant’s left (Fig. 1). Participants placed their hands on each side of the table. A small red sticker was put on the table at 12 cm left from the mirror and 30 cm in front of the table’s edge to mark the participants’ left index finger placement. A graph paper was placed on the participant’s right-hand side of the table to record reaching positions. For the foot condition, a 60 cm × 90 cm mirror was positioned vertically on the floor (Fig. 2). Participants sat at the edge of the mirror, placing it between their legs. Their left foot (the first digit) was placed on a red sticker at 12 cm left from the mirror and approximately 50 cm in front of the participant’s abdomen. For both conditions, participants were allowed to see only on their left side (reflective surface side), while their right side was hidden behind the mirror. The reaching target line (12 cm right from the mirror) was indicated in the mirror as a white line (as if it were drawn on the hidden side), which was actually the mirror-reflection of a line drawn 12 cm left from the mirror (Fig. 1, bottom right panel). To prevent the mirror-reflected hand (or foot) from interfering with reaching by the hidden right hand (or foot), it was made invisible by a black cloth when they reached the target line (cloth size: 18 cm × 45 cm (hand condition), 22 cm × 90 cm (foot condition). As the cloth concealed only the nearest part of the mirror to participants, they could see approximately half of the target line. The distance of the right hand (or foot) from the participant’s coronal place was consistent with that of the left hand (or foot).

### Procedure

At the beginning of the hand session, the participants practiced reaching the target line in a few trials with their unseen right hand, while the mirror reflection of the left hand was occluded by the cloth. The experimenter placed the participant’s right hand at the start positions (somewhere between 5 and 30 cm right from the mirror). The participants received feedback on reaching accuracy by directly looking at the right-hand side. After two more practice trials without feedback at 19 cm and 5 cm start positions, the baseline condition began, followed by the immediate or delay condition. In the mirror illusion induction, the start position of the right hand was always 19 cm to the right of the mirror (uncovered). The participants were asked to see the mirror reflection of their left hand and tap the index fingers of both hands synchronously ten times at around 2 Hz. Immediately after the tapping, the mirror reflection of the left hand was covered by the cloth (but the target line could be seen). While the participants reached the target line soon after this procedure in the immediate condition, they did so 15 s later in the delay condition. The experimenter verbally gave the go signal in both conditions. After the reaching, the experimenter marked the reaching position (tip of the right index finger) on the graph paper using a pen. In between the trials, the experimenter swung the participant’s right hand side-to-side before placing it on the start position (19 cm to the right of the mirror) so that the participant was not given information regarding their reaching accuracy. Following the removal of the cloth from the mirror, the next trial began. After completing the experimental session, the participants were asked to rate how much they perceived the mirror reflection of the left hand as their right hand (i.e., ownership score) ranging from 0 (not perceived as the right hand at all) to 100 (definitely perceived as the right hand). For the foot session, the first digits of the left and right feet were used instead of the index fingers of the left and right hands. Both toes were tapped during the illusion induction. Besides this, the procedure was the same as in the hand session.

### Assessment of fall risk and cognitive functions

This study used the TUG test to investigate the relationship of visuo-proprioceptive processing in localization of the body parts with participants’ risk of falling. This test is commonly employed as a clinical tool to identify older adults at risk of falling^39^. The participants were asked to stand up from a standard chair with a seat height of approximately 40 cm, walk as quickly as possible to a marker placed at 3 m, turn around the marker, walk back to the chair, and sit down. The time between standing up to being re-seated was measured with a stopwatch. Further, the Trail Making Tests A and B (TMT-A and TMT-B) assessed the participants’ executive functions, including attentional control and task switching^40,41^, because studies have suggested that declines in executive and attentional systems could impact multisensory processing^42^.

### Statistical data analysis

Statistical analyses and visualizations were performed using R software (4.0.5)^43^ with tidyverse^44^ and anovakun^45^ packages. The reaching positions of the right index finger (and the first digit of the right foot) were recorded only in the horizontal dimension (i.e., the distance from the mirror). Each participant’s reaching position was the average across four trials for the immediate and delay condition, while that for the baseline condition was the average across two trials of a 19-cm start position (Supplement Table S1 shows differences in reaching positions between two start positions in the baseline condition). The reaching errors were calculated by subtracting reaching positions in the baseline condition from those in the immediate and delay conditions. Negative and positive values indicate the reaching errors in the direction of the mirror and its opposite direction, respectively. Positive errors suggest visual capture effects by the mirror-reflection. Shapiro–Wilk tests revealed that the reaching data in each condition were normally distributed. A one sample *t*-test against zero was performed in each condition to investigate whether the reaching error occurred. Then, a two-way mixed-design analysis of variance (ANOVA) was performed for each body part with one between-participants factor (Age) and one within-participant factor (Reaching). The significance level alpha was set at 0.05 and marginal significance level was set between 0.05 and 0.10. However, as this study focused on whether the change in reaching performance across time differed between the age groups, the simple effects of Reaching and Age were tested even when the interaction was not significant (planned comparison). Mendoza's Multisample Sphericity Test assessed whether the assumption of sphericity was met for ANOVA^46^. When the assumption of sphericity was violated, the degree of freedom was adjusted by using Greenhouse–Geisser’s epsilon. Regarding ownership score, a Mann–Whitney test for each body part condition investigated the effect of Age, as the Shapiro–Wilk tests revealed the violation of normal distribution.

For older adults, correlation analyses were exploratorily performed to investigate the association of reaching performance with fall risk (TUG) and cognitive functions (TMT-A and TMT-B). Specifically, the reaching performance variables included reaching error in the immediate and delay conditions and how much the reaching error changed over time (temporal shift). The temporal shift was computed by subtracting the reaching position in the delay condition from that in the immediate condition. Positive and negative values represent shifts toward and away from the mirror, respectively. Spearman’s correlation coefficients were calculated.

## Results

### Reaching performance and ownership score

**Figure 3:**
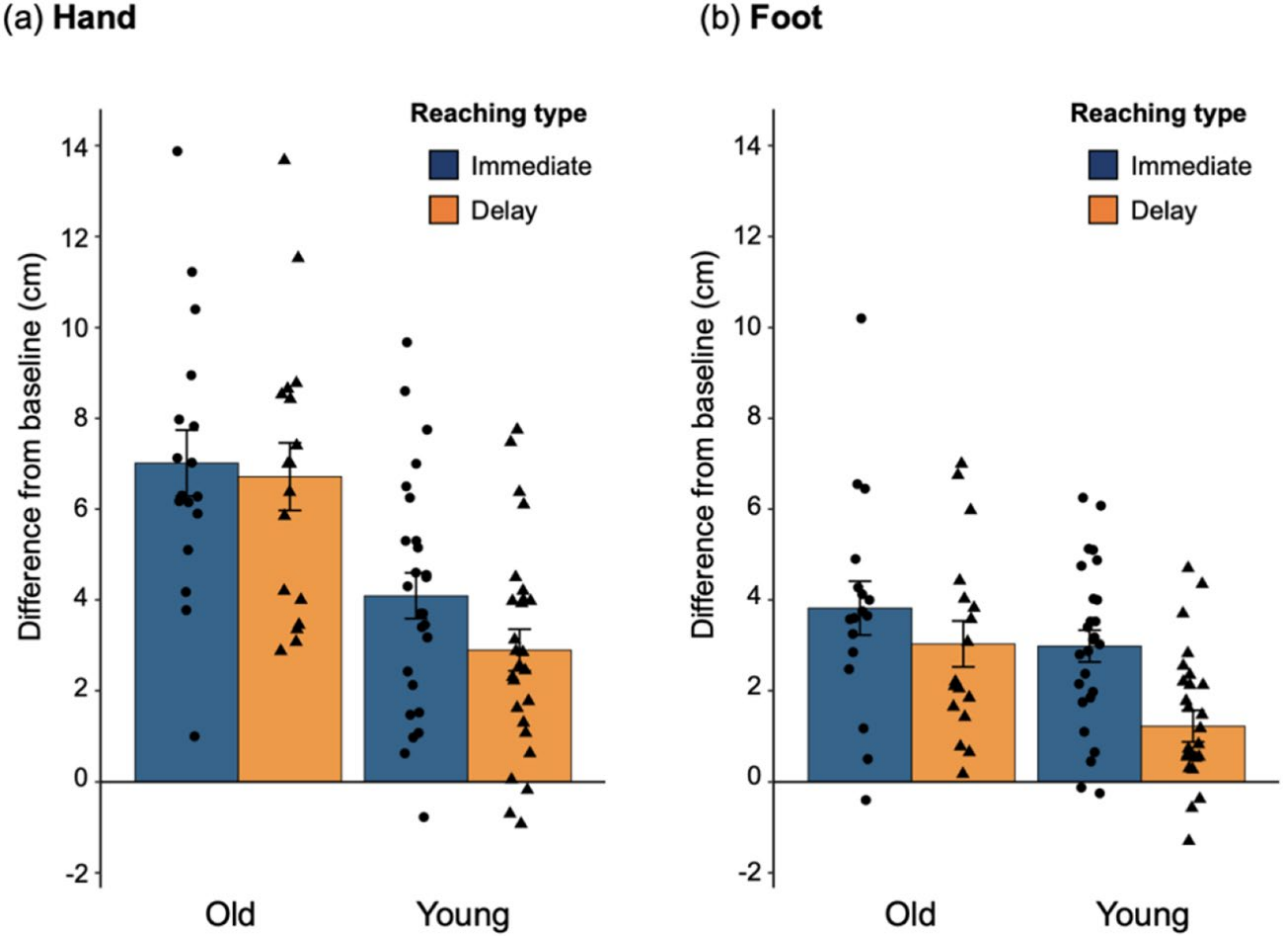
Reaching error in the hand and foot conditions for older (*N* = 17) and young adults (*N* = 26). Reaching errors were calculated by subtracting reaching positions in the baseline condition from those in the immediate and delay conditions. Negative and positive values indicate the reaching errors in the direction of the mirror and its opposite direction, respectively. Dots indicates individual data. Error bars denotes the standard error of the mean. Plots were generated using R software version 4.0.5 (R Core Team (2021). R: A language and environment for statistical computing. R Foundation for Statistical Computing, Vienna, Austria. http://www.R-project.org/).

One sample *t*-tests revealed significant positive reaching errors in all conditions for both young (*t* (25) > 3.51, *p* < 0.002, *d* > 0.68) and older adults (*t* (25) > 6.00, *p* < 0.001, *d* > 1.45), indicating that the mirror illusions occurred in both groups irrespective of body part (hand and foot) and reaching type (immediate and delay; Fig. 3; Supplementary Figure S1 shows the absolute reaching position data).

In the hand condition, a two-way ANOVA revealed significant main effects of Age [*F* (1, 41) = 17.65, *p* < 0.001, η^2^ = 0.277] and Reaching [*F* (1, 41) = 8.88, *p* = 0.005, η^2^ = 0.014] and a marginally significant interaction effect [*F* (1, 41) = 3.18, *p* = 0.082, η^2^﻿ = 0.005]. For the planed analysis for the interaction effect, a simple effect of Age was significant in both reaching conditions: older adults exhibited larger reaching error than young adults [immediate: *F* (1, 41) = 11.67, *p* = 0.002, η^2﻿^ = 0.222; delay: *F* (1, 41) = 21.42, *p* < 0.001, η^2^﻿ = 0.343]. Regarding the simple effect of Reaching, the reaching error in the delay condition significantly decreased compared to the immediate condition in young adults [*F* (1, 25) = 11.86, *p* = 0.002, η^2﻿^ = 0.058], but not in older adults [*F* (1, 16) = 0.88, *p* = 0.326, η^2﻿^ = 0.003].

In the foot condition, a two-way ANOVA revealed all the significant main and interaction effects [Age: *F* (1, 41) = 5.29, *p* = 0.027, η^2^﻿ = 0.090; Reaching: *F* (1, 41) = 30.29, *p* < 0.001, η^2^﻿ = 0.084; Age × Reaching: *F* (1, 41) = 4.40, *p* = 0.042, η^2﻿^ = 0.012]. Regarding the planed analysis for the interaction effect, a simple effect of Age was significant in the delay condition [*F* (1, 41) = 9.27, *p* = 0.004, η^2^﻿ = 0.185], but not in the immediate condition [*F* (1, 41) = 1.68, *p* = 0.202, η^2﻿^ = 0.039]. Regarding the simple effect of Reaching, the reaching error in the delay condition significantly decreased compared to the immediate condition in young adults [*F* (1, 25) = 38.62, *p* < 0.001, η^2﻿^ = 0.202], but not in older adults [*F* (1, 16) = 4.42, *p* = 0.052, η^2^ = 0.031]. Thus, while the reaching errors were compatible for both age groups immediately after the illusion induction, the error decreased over time only for the young adult group.

The ownership scores of the mirror image were significantly larger for older adults (hand: 84.12 ± 32.42; foot: 84.12 ± 32.42) than younger adults (hand: 76.92 ± 17.89; foot: 73.85 ± 19.10) (hand: *W* = 329.5, *p* = 0.005; foot: *W* = 340.5, *p* = 0.002).

### Correlation between reaching performance and fall risk or cognitive abilities

The temporal shift was significantly correlated with the TUG times (hand: *r* = − 0.503, *p* = 0.040; foot: *r* = − 0.535, *p* = 0.027): older adults with shorter TUG times (i.e., lower risk of falling) exhibited a larger reduction in reaching position across time. The other correlations were not significant (Table 2).

**Table 2:** Correlation coefficients between reaching error and TUG and between reaching error and TMT-A/B.

	Mean (SD)	Hand			Foot		
		Immediate	Delay	t. shift	Immediate	Delay	t. shift
TUG	6.67 (1.01)	− .098	.084	− .503*	− .071	.189	− .535*
TMT-A	49.18 (13.52)	− .092	− .088	.234	− .392	− .371	.175
TMT-B	78.00 (29.25)	.361	.418	.050	− .103	.075	− .322

t. shift: temporal shift in reaching error between the immediate and delay conditions.

*p < .05.

## Discussion

This study used the mirror hand/foot illusion to investigate whether visuo-proprioceptive processing in the sense of body position was changed with age, body part, and delay from illusion induction, and whether it was associated with older adults’ fall risk. There were three main results: (1) Older adults exhibited larger reaching errors than young adults in the hand condition and in the delay condition of the foot; (2) The reaching error decreased over time after the illusion induction in young adults but was almost constant in older adults, regardless of the tested body part; (3) Older adults with lower TUG performance exhibited less changes in reaching performance across time compared to those with higher TUG performance. This suggests that age-related change in visuo-proprioceptive processing in the sense of body position depends on the body part and delay from the occlusion of vision. This also suggest that the changes in multisensory processing are associated with declines in physical or cognitive functions related to fall risk.

In Holmes et al.^3^, the reaching error was approximately 3 cm when the distances matched those in this study (i.e., the mirror reflected hand and target was positioned at 12 cm right from the mirror and the unseen hand was at 19 cm). The reaching error was compatible with that in this study (2.95 ± 0.40 cm in the immediate hand condition), although there were several differences in experimental methods between Holmes et al.^3^ and this study. This suggests the validity of this study’s experimental procedure.

This study, using the mirror illusion, showed that older adults exhibited a larger mirror illusion in the hand condition than young adults, suggesting a greater reliance on visual over proprioceptive information. This is inconsistent with previous studies on rubber hand illusion^11–15^, but consistent with several studies investigating the effect of occlusion of vision on reaching trajectory, which have shown that older adults rely more on visual than proprioceptive information compared with younger adults^17,18^. Thus, the rubber hand illusion may be less sensitive in investigating the age-related change in visuo-proprioceptive processing in perceived body position. While the prosthetic hand is presented in the rubber hand illusion, participants’ real hand is presented in the mirror hand illusion. Moreover, studies showed that the appearance of the presented hand had an impact on the experience of the rubber hand illusion^19^ and mirror hand illusion^21^. Thus, the difference in the appearance of the presented hands might affect the localization performance. Alternatively, a difference in the measuring method for the perceived position of the body parts might influence the results. Most previous studies investigating age-related differences in rubber hand illusion have utilized perceptual or motor responses to localize the illusion-induced unseen hands, such as a ruler placed above the unseen hand^47^, a mouse cursor or ruler navigated by participants^14,15^, or pointing with the contralateral, non-illusion induced hand^11–13^. However, no study has used reaching or pointing by the unseen hand. Previous studies show that illusion induction by participants’ own movements selectively affected manual responses but not perceptual responses, suggesting that distinct mechanisms are involved in perceptual and motor body representations^22–24^. According to this study, the motor body representation might be more susceptible to the aging effect than perceptual body representation. Future studies should design experiments using both perceptual and motoric measures at the same time.

Contrastingly, the age-related difference was not observed in the immediate condition in the foot condition. Studies have shown a decrease in gait function with increasing age^33^. Therefore, this study predicted that proprioceptive information from the lower limb might deteriorate more than that from the other body parts in older adults so that visual information had a stronger influence on position sense in the perceived foot position. However, the results were not consistent with this prediction. Hide et al.^34^ reported that older adults with higher TUG performance hardly experienced the rubber *foot* illusion, indicating that visual information had little influence in some older adults. The current older adult sample exhibited relatively higher TUG performance (6.67 ± 1.01 s) than those in Hide et al. (mean across all older adults: 7.35 ± 1.65 s; high TUG performance older adults: 6.08 ± 0.47 s; low TUG performance older adults: 8.61 ± 1.40 s). Thus, older adults in this study might rely less on vision than proprioception. Nevertheless, a larger number of older adult participants should be tested to conclude this issue. Alternatively, no salient age-related effect in the immediate, foot condition might be due to the displacement size between the mirror-reflected and unseen hands. While the physical displacement size was the same irrespective of the body part, the displacement size in visual angle was smaller in the foot than hand condition because the foot position was distant from the eyes. Holmes et al.^3^ reported that the visual influence on hand position perception was more salient with an increase in displacement size. Thus, the displacement size was possibly not enough to induce age-related differences in multisensory processing in perceived foot position.

### Different time courses in weighting recalibration between young and older adults

Bellan et al.^4^ investigated how visuo-proprioceptive processing in hand position changed across time after the removal of visual information. The results showed that, while the unseen hand was initially localized at the last seen position, it was gradually localized toward the proprioceptively-defined, physical position of the hidden hand across time. This suggests that the trace of the visually-defined hand position remains for a while even after the removal of visual inputs about the hand but gradually decays over time. They also measured the localization performance after the exposure to the *consistent* visuo-proprioceptive information in the hand position. Results showed that the localization was shifted in the same direction as that in the inconsistent condition (i.e., outward from the body midline) but was more gradual and slow. Thus, the authors suggest that, while the gradual shifts in the consistent condition are due to proprioceptive drift reported by several studies^48,49^, the quicker shifts in the inconsistent condition could include both proprioceptive drift and gradual reweighting of visual and proprioceptive information or spontaneous decay of new mapping between the visual and proprioceptive information constructed during the exposure. Typical proprioceptive drift reportedly occurs in the outward direction from the midline^50,51^, 30 s after the removal of visual information^52^. However, in this study, participants reached the target within 30 s, and the reaching positions were in the direction of the mirror (to the midline of the body). Thus, the shift in reaching performance across time in young adults in this study cannot be fully explained by typical proprioceptive drift phenomena. As Bellan et al.^4^ suggest, reweighting of visual and proprioceptive information could occur in this study because the trace of visual information gradually decays so that the reliability of visual information is degraded relative to that of proprioceptive information. Alternatively, per the prism adaptation study^53^, the new mapping between visual and proprioceptive signals constructed during the exposure to their inconsistency may spontaneously decay after the removal of visual information.

Unlike young adults, the reaching performance did not change over time in older adults irrespective of the body part. Previous studies show that older adults cannot flexibly adjust the weighting of information from several sensory modalities when sensory inputs are suddenly changed in reaching^25^ and postural control^26^. The present results are consistent with these studies. Further, this study’s results shows that older adults with better TUG times exhibited a more similar reaching performance to young adults, specifically, greater reduction of reaching errors across time than those with slower TUG times. Studies also show the association between TUG or gait functions and several cognitive abilities^54,55^ or multisensory processing^34–36,56^. Considering the correlation in both hand and foot conditions (although correlations in a small sample size must be interpreted with caution), some physical and cognitive functions related to TUG may be associated with the overall perceptual functions for monitoring the external environment and appropriately reweighting sensory information in accordance with the environment.

This study used the reaching procedure to investigate visuo-proprioceptive processing in the perceived position of the body parts according to previous studies on the mirror illusion^3^. Thus, the perceived position of the right hand or foot was indirectly estimated by the reaching performance instead of directly asking where the body part was by pointing by the left hand or mouse cursor. The rationale is that the reaching should be shifted if the perceived position is biased in either direction. Indeed, previous studies reported a correlation between the perceived initial hand position and reaching error^3^ and that the reaching errors were systematically biased according to the visually-defined hand position^20^. However, because these studies targeted only young adults, it is unclear whether the same correlation is observed in older adults. Dynamic position information during reaching may modulate reaching performance in older adults; this should be addressed in future studies. Nevertheless, as older adults in this study hardly moved their body parts in the immediate and delay condition, especially in the hand condition, dynamic position information obtained during reaching would be unlikely to influence reaching performance.

## Conclusions

This study investigated the age-related changes in visuo-proprioceptive processing in perceived body position using mirror hand/foot illusions. The results suggested that older adults, as opposed to younger adults, relied more on visual over proprioceptive information in the hand condition, but not always in the foot condition. The absence of age-related differences in the immediate foot condition might be due to the high functional capacity of lower limbs in older adults of this study or relatively small visuo-proprioceptive inconsistency in visual angle presented in the foot condition. Irrespective of the body part, the reaching positions were constant across time in older adults, but gradually shifted to the accurate position in young adults. This was more salient for older adults with higher risk of falling than those with a lower risk. This suggests that older adults, especially those with a relatively high risk of falling, face difficulties in appropriately processing visual and proprioceptive information for body perception based on changes in the external environment.

## Supplementary Information

Supplementary Information.

## Data Availability

The datasets generated and analyzed during the current study are available from the corresponding author on reasonable request.
