# Supplementary material for: Age-related changes in visuo-proprioceptive processing in perceived body position
Source: Sci Rep. 2022 May 18;12:8330. doi: 10.1038/s41598-022-12022-w (PMC9117257; doi:10.1038/s41598-022-12022-w)
Supplement: Supplementary file 1 — Supplementary Information. [file 41598_2022_12022_MOESM1_ESM.docx]

**Age-related changes in visuo-proprioceptive interactions in perceiving body position**

**Wataru Teramoto**

Department of Psychology, Graduate School of Humanities and Social Sciences, Kumamoto University, 2-40-1 Kurokami, Kumamoto, 860-8555 Japan

**Supplementary Table S1.** **Mean reaching position in each start position for the baseline condition (cm)**

*Note*. Values enclosed in parentheses represent standard deviation of the mean. Asterisks repserent significant difference in reaching position between two start positions revealed by paired *t*-tests (*: *p* < .05).


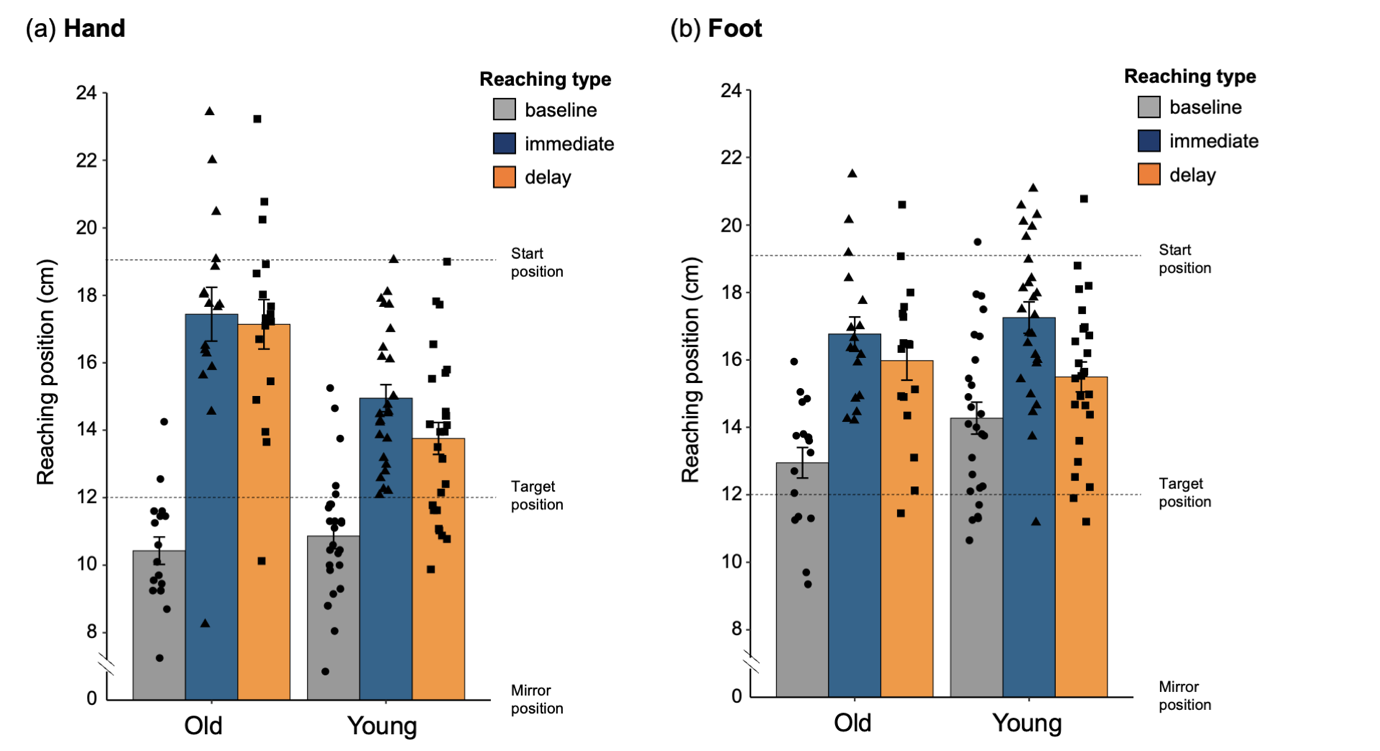


*Supplementary Figure S1*. Reaching position in the hand and foot conditions for older (*N* = 17) and young adults (*N* = 26). Zero represents the mirror position. Thin horizontal lines represent the start position (19 cm right from the mirror) and target position (12 cm right from the mirror). In all plots, dots indicates individual data. Error bars denotes the standard error of the mean.

Two separate two-way ANOVA at each body part level were performed. The ANOVAs revealed a significant interaction of Age × Reaching in both body parts [hand: *F* (1.54, 62.97) = 14.37, *p* < .001, eta^2 = .050; foot: *F* (2, 82) = 5.00, *p* = .009, eta^2 = .019]. In the hand condition, the simple effect analysis of Age revealed that reaching position was significantly larger for older adults than young adults in the immediate [*F* (1, 41) = 9.41, *p* = .004, eta^2 = .187] and delay conditions [*F* (1, 41) = 16.58, *p* < .001, eta^2 = .288], but not in the baseline condition [*F* (1, 41) = 0.58, *p* = .449, eta^2 = .013]. This indicates that a larger displacement of reaching occurred for older than younger adults, while the baseline condition was the same between the age groups. Contrastingly, in the foot condition, the simple effect analysis of Age did not reach significance in any Reaching conditions [*F* (1, 41) < 3.65, *p* > .063, eta^2 < .082]. This indicates that the reaching performance was almost the same between the age groups irrespective of the Reaching conditions.

The simple effect of Reaching was also significant in both groups for the hand [young: *F* (2, 50) = 45.50, *p* < .001, eta^2 = .403; older: *F* (1.29, 20.64) = 79.60, *p* < .001, eta^2 = .595] and foot conditions [young: *F* (2, 50) = 41.69, *p* < .001, eta^2 = .219; older: *F* (2, 32) = 32.64, *p* < .001, eta^2 = .389]. The multiple comparison of the simple effect (Holm-Bonferroni method) revealed that the immediate and delay conditions were larger than the baseline condition [young: *t* (25) > 3.51, *p* < .002; older: *t* (16) > 5.99, *p* < .001], regardless of the body part, indicating that the mirror illusions occurred in both groups. Further, the reaching position was significantly decreased (i.e., approached the mirror or target line) in the delay condition compared with the immediate condition in young adults [hand: *t* (25) = 6.33, *p* < .001; foot: *t* (25) = 6.21, *p* < .001], while it was constant over time in older adults [hand: *t* (16) = 0.36, *p* = .362; foot: *t* (16) = 2.10, *p* = .052].
